# Personalizing Inhaled Therapy in COPD and Asthma: Integrating Drug, Device, and Patient Factors

**DOI:** 10.3390/biomedicines14102184

**Published:** 2026-09-26

**Authors:** Kenneth Yung, Shih-Lung Cheng

**Affiliations:** 1 Department of Internal Medicine, Far Eastern Memorial Hospital, New Taipei City 220, Taiwan; femhf0277@femh.org.tw; 2 Department of Chemical Engineering and Materials Science, Yuan Ze University, Taoyuan 320, Taiwan

**Keywords:** inhaler devices, pharmaceutical aerosols, COPD, asthma, small airway disease, peak inspiratory flow rate

## Abstract

The management of chronic obstructive pulmonary disease (COPD) and asthma depends largely on the effective delivery of aerosolized medications to the respiratory tract. Clinical efficacy is determined not only by the active pharmaceutical ingredient but also by the interplay among drug formulation, aerosol particle size, inhaler device characteristics, dosing frequency, and patient-specific factors. This review examines the major inhaled therapeutic platforms, including soft mist inhalers (SMIs), pressurized metered-dose inhalers (pMDIs), and dry powder inhalers (DPIs), and evaluates how their characteristics influence pulmonary drug delivery. We examine the impact of particle size and extrafine formulations on peripheral deposition and small airway disease (SAD), as well as clinically relevant factors including peak inspiratory flow rate (PIFR), inhaled corticosteroid-associated pneumonia risk, dosing frequency and adherence, rescue inhaler requirements, inhaler technique, cost and accessibility, and environmental impact. By integrating inhaler characteristics with real-world clinical considerations, this article provides a structured, evidence-based framework for individualized inhaler selection in obstructive lung disease. However, the available evidence is limited by heterogeneity across devices, formulations, study populations, and assessment methods, highlighting the need for further comparative effectiveness studies and evaluation of emerging inhaler technologies.

## 1. Introduction

Chronic Obstructive Pulmonary Disease (COPD) and asthma represent a significant global health burden, ranking among the leading causes of morbidity and mortality worldwide [1]. The goal of medical therapy in these patients is to alleviate symptoms, improve lung function, and reduce the frequency and severity of acute exacerbations, while minimizing the adverse effects that may be associated with therapy.

Inhalation therapy is the foundation of pharmacologic treatment for both diseases, as it allows direct delivery of bronchodilators and corticosteroids to the airways while minimizing unwanted systemic exposure and associated adverse effects. However, unlike oral or intravenous drug administration, the efficacy of inhaled therapy depends not only on the active pharmaceutical ingredient. Drug formulation, aerosol characteristics, delivery-device engineering, and the patient’s ability to use the device effectively all influence pulmonary drug deposition and ultimately treatment effectiveness.

Advances in aerosol science and inhaler technology have resulted in a diverse range of therapeutic options, including pressurized metered-dose inhalers (pMDIs), dry powder inhalers (DPIs), and soft mist inhalers (SMIs), with substantial differences in aerosol generation and delivery characteristics. At the same time, inhaled therapies differ in their pharmacologic properties, formulation, particle size, dosing frequency, and safety profiles. Consequently, selecting an inhaled therapy requires consideration not only of the prescribed drug or inhaler platform, but also of how these characteristics interact with the individual patient.

This review first introduces key principles of aerosol science and pulmonary drug deposition and examines how inhaler devices and formulations influence drug delivery. Building on this foundation, we evaluate clinically relevant factors that may guide individualized treatment selection, including inspiratory capability, small airway disease, inhaled corticosteroid-associated pneumonia risk, dosing frequency and adherence, rescue inhaler requirements, inhaler technique, cost and accessibility, and environmental impact. By integrating these pharmacologic, device-related, and patient-specific considerations, we aim to provide a practical framework for personalizing inhaled therapy in asthma and COPD.

## 2. Literature Search and Evidence Selection

This narrative review was developed using an iterative, topic-based approach to literature identification. Relevant publications were identified through searches of PubMed/MEDLINE, review of major international guidance documents including GINA and GOLD, and examination of reference lists from relevant reviews and key studies. Searches were conducted according to the individual topics addressed in the manuscript, including inhaler-device characteristics, aerosol deposition, particle size, inspiratory flow, inhaler technique, adherence, treatment selection, cost and accessibility, and environmental considerations.

Studies were selected on the basis of their relevance to the specific clinical or formulation question under discussion. Priority was given to current guidelines, systematic reviews and meta-analyses, randomized clinical trials, clinically relevant observational studies, and aerosol deposition or formulation studies directly relevant to inhaler performance. Literature published through August 2026 was considered, without a predefined lower date limit because older studies were retained when they provided foundational evidence regarding aerosol behavior or inhaler-device principles.

## 3. Foundations of Aerosol Science and Drug Delivery

The primary objective of inhalation therapy is to deliver bronchodilators and corticosteroids to their target regions within the respiratory tract while minimizing oropharyngeal deposition and systemic absorption. However, the clinical translation of aerosol science requires an understanding of how aerosol properties influence pulmonary deposition.

Several related measures of particle size should be distinguished. Geometric particle diameter describes the physical dimensions of a particle, whereas aerodynamic diameter describes its behavior while suspended in air and is therefore influenced not only by geometric size, but also by particle density and shape [2]. The mass median aerodynamic diameter (MMAD) is a commonly used metric to characterize the midpoint of an aerosol particle’s size distribution by mass. MMAD is an important determinant of regional deposition, although deposition is also influenced by airflow, inhalation pattern, airway anatomy, and disease.

Deposition is governed by three primary mechanisms, summarized in Table 1.

Inertial impaction affects larger, high-velocity particles that cannot follow changes in airflow direction, leading to deposition at airway bends. This mechanism mainly affects particles with aerodynamic diameters greater than approximately 5 μm. Larger particles deposit most in the upper respiratory tract (oropharynx) and the first generations of the tracheobronchial tree. Impaction is strongly velocity dependent and explains why excessively rapid inhalation or high spray velocities increase oropharyngeal deposition [3]. This is particularly relevant for conventional pMDIs which have higher spray velocities.

Conversely, particles with an aerodynamic diameter below approximately 5 μm are less susceptible to inertial impaction in the upper airways and can penetrate deeper into the distal bronchi [3]. Accordingly, the fine particle fraction (FPF), defined as the proportion of the emitted aerosol mass comprising particles with an aerodynamic diameter below a specified threshold, commonly 5 μm, represents the fraction with a greater probability of bypassing the upper airway and reaching the lower respiratory tract [4].

Particles in the 1–5 μm range are capable of penetrating into the lower respiratory tract, where gravitational sedimentation, the time-dependent settling of aerosols under gravity, becomes increasingly important. Breath holding increases dwell time in the airways, thereby enhancing deposition by sedimentation [3]. Particles smaller than 1 μm may penetrate into the most distal lung regions, although a substantial fraction may remain suspended and subsequently be exhaled. For these ultrafine particles, Brownian diffusion—resulting from random molecular motion that causes particles to collide and deposit on the airway wall—becomes the predominant mechanism of deposition [5]. Adequate breath-hold duration increases the opportunity for deposition before particles are exhaled.

Based on the above physical principles, inhalation pattern influences regional aerosol deposition. Slower inhalation reduces inertial impaction and promotes sedimentation, whereas breath holding increases the time available for gravitational deposition. However, the optimal inhalation maneuver is device-dependent.

Particle properties beyond geometric diameter can also influence aerosol behavior and pulmonary deposition. Aerodynamic diameter is affected by particle density and shape; therefore, particles of similar geometric size may behave differently in the airways. Particle morphology can also influence cohesion and deaggregation, particularly in dry-powder formulations. Hygroscopicity, the tendency of particles to absorb water, may increase particle size within the humid respiratory tract and thereby alter deposition. Thus, pulmonary deposition depends on overall aerodynamic behavior rather than geometric particle diameter alone [6]. Formulation-specific MMAD values for representative inhaler products are summarized in Appendix A, Table A1.

## 4. Measuring Drug Deposition in the Respiratory Tract

The most common method for quantification of inhaled drug deposition uses nuclear imaging, in which the inhaled formulation is radiolabeled with a radioisotope and pulmonary deposition is assessed after inhalation. Conventional planar gamma scintigraphy enables direct in vivo measurements of total lung deposition, extrathoracic deposition, and regional distribution within the lungs. However, planar gamma scintigraphy has limited spatial resolution and cannot fully characterize the three-dimensional distribution of deposited aerosol [7]. Three-dimensional nuclear imaging techniques, including single-photon emission computed tomography and positron emission tomography, can provide tomographic assessment of deposition and better characterize regional and three-dimensional heterogeneity within the lungs. Limitations of nuclear imaging include the need for radiolabel modification of a product, specialized imaging facilities, and exposure to ionizing radiation [8].

Functional Respiratory Imaging (FRI), by contrast, is a computational technique that integrates high-resolution computed tomography-derived three-dimensional airway reconstructions with fluid dynamic modeling to simulate airflow and particle transport. Advanced computational models are used to predict intrathoracic deposition patterns and can generate detailed maps of drug distribution. Input variables such as particle size distribution, drug plume characteristics and inhalation flow can be modified to model multiple inhalation scenarios without repeated human exposure or radiolabeling [9]. In vivo nuclear imaging and FRI therefore provide complementary approaches to evaluating inhaler performance and drug distribution.

## 5. Impact of Particle Size and Lung Deposition on Outcomes

Studies of extrafine hydrofluoroalkane (HFA)–beclometasone formulations provide a practical example of how the same active ingredient, when engineered to a smaller particle size, influences drug delivery and clinical response. Older chlorofluorocarbons (CFC)-based beclometasone suspension formulations have a mass median aerodynamic diameter (MMAD) of approximately 3.5–4.0 µm. Reformulation to an HFA solution produces an extrafine aerosol, reducing the MMAD to about 1.1 µm.

Radiolabeled imaging studies demonstrate substantially higher lung deposition with extrafine HFA–beclometasone (approximately 56%) compared with CFC–beclometasone (approximately 16%), along with reduced oropharyngeal deposition [10]. Dose–response studies indicate that the systemic exposure of equivalent nominal doses of extrafine HFA–beclometasone is approximately 1.7-fold higher than that of CFC–beclometasone, consistent with its substantially greater pulmonary delivery [11]. Correspondingly, subsequent trials demonstrate that extrafine HFA–beclometasone achieves comparable asthma control at approximately half the daily dose of CFC–beclometasone [12]. Extrafine HFA–beclometasone has also been associated with reduced air trapping on functional computed tomography [13].

The combination of maintained efficacy at lower doses and reduced oropharyngeal deposition has potential implications for safety and tolerability. In one study, 800 µg HFA–beclometasone and 1600 µg CFC–beclometasone produced similar improvements in lung function and asthma symptoms, while fewer patients receiving extrafine HFA–beclometasone had plasma cortisol levels below the normal range (5.1% vs. 17.3%), suggesting less adrenal suppression [14]. A key limitation of these comparisons is that the transition from CFC to HFA formulations altered not only particle size, but also formulation and inhaler characteristics, making it difficult to isolate the independent effect of particle size.

The effect of particle size was more directly evaluated in a study by Usmani et al., in which albuterol was delivered as aerosols with three distinct particle sizes (1.5, 3, and 6 µm MMAD) at the same nominal dose. Deposition was quantified using γ-scintigraphy and bronchodilator response was assessed [15]. Smaller particles achieved greater total lung deposition (1.5 µm: 56%, 3 µm: 50%, 6 µm: 46%) and increased peripheral deposition (25%, 17%, and 10%, respectively), whereas larger particles showed higher oropharyngeal deposition (15%, 31%, and 43%). Despite this more favorable deposition profile, smaller particles did not produce a superior bronchodilator response. Instead, bronchodilation increased with particle size, with FEV_1_ improvement following the order 6 µm > 3 µm > 1.5 µm (ΔFEV_1_: 551 ± 221 mL, 457 ± 200 mL, and 347 ± 172 mL, respectively). The authors proposed that this reflects more effective targeting of conducting airway smooth muscle where β_2_-receptor–mediated bronchodilation predominantly occurs.

Taken together, these findings demonstrate that particle size predictably influences total and regional lung deposition, but greater peripheral deposition does not necessarily translate into superior clinical efficacy. The clinical consequences depend on where the relevant pharmacological target is located, the drug being delivered, the formulation and device, and the underlying disease phenotype. Thus, a smaller MMAD should not be interpreted as inherently indicating a more effective inhaled therapy. The potential relevance of enhanced peripheral deposition in patients with small airway dysfunction is discussed further in Section 7.3. Representative study-specific values for pulmonary and extrapulmonary drug deposition with selected inhaler devices are summarized in Table 2**.** Commercial device names are provided for identification of specific inhaler platforms; however, availability and brand names may vary by country or region.

## 6. Engineering Differences of Inhaler Devices

Among handheld inhaler devices, the three major device families are pressurized metered-dose inhalers (pMDIs), dry powder inhalers (DPIs), and soft-mist inhalers (SMIs). Contemporary devices have been engineered to overcome the limitations of earlier inhaler systems, including the environmental concerns with CFC propellants, and the higher spray velocities and droplet sizes associated with greater oropharyngeal deposition [21]. The principal characteristics of these device classes are discussed below, with their major advantages, limitations, and patient factors relevant to device selection summarized in Table 3.

### 6.1. Pressurized Metered-Dose Inhalers (pMDIs)

pMDIs are propellant-driven systems that deliver a fixed metered volume of drug via a high-velocity aerosol plume generated by liquefied propellants. Advantages of pMDIs include compact design, rapid delivery, relative independence from patient inspiratory effort, and broad compatibility with combination formulations, including ICS/long-acting β_2_-agonist (LABA), LABA/long-acting muscarinic antagonist (LAMA), and single-inhaler triple therapy. However, pMDIs require coordination between actuation and inhalation, which is a major limitation in those with impaired dexterity or cognitive dysfunction [26]. Suboptimal technique promotes inertial impaction in the oropharynx due to high initial plume velocities. The addition of a valved holding chamber (VHC) can improve lung delivery and reduce oropharyngeal deposition, but requires carrying an additional device [27].

Dose consistency in pMDIs is influenced by formulation characteristics and handling technique. In solution-based pMDIs, the drug is dissolved in the propellant, making the emitted dose relatively insensitive to shaking. In suspension-based pMDIs, the drug is present as solid particles dispersed within the propellant, and inadequate shaking or prolonged delay between shaking and actuation can therefore increase variability in the emitted dose. Delayed actuation after shaking has been shown to substantially affect dose delivery from suspension formulations, whereas solution formulations remain comparatively stable [28,29]. In one study, emitted doses from suspension formulations ranged from 32% to 380% of the labeled claim [29].

Several advances have been developed to address some of the limitations of pMDIs. Aerosphere Co-suspension Technology, used in pMDI formulations of budesonide/glycopyrronium/formoterol and glycopyrronium/formoterol, represents one such approach. In this system, porous phospholipid-based particles are co-suspended with active drug crystals, reducing aggregation and sedimentation and improving dose uniformity, with relatively stable delivery throughout inhaler life despite variations in shaking or shake-to-actuation delay [30,31].

Breath-actuated pMDIs address another limitation of conventional pMDIs by reducing the need for hand-breath coordination. Rather than requiring the patient to manually actuate the canister, these devices use inspiratory effort to trigger aerosol release. Because aerosol generation remains propellant-driven, they require less inspiratory flow than DPIs, although sufficient effort is still needed to activate the device [32].

A further advance is the development of extrafine formulations, such as extrafine beclometasone dipropionate/formoterol and beclometasone dipropionate/formoterol/glycopyrronium. These solution-based formulations are able to generate particles with an MMAD below 2 μm, thereby increasing the potential for peripheral airway deposition [30].

### 6.2. Dry Powder Inhalers (DPIs)

Dry powder inhalers are propellant-free, breath-actuated devices in which inspiratory effort disperses drug particles into a respirable aerosol. One advantage of DPIs is that they do not require hand-breath coordination. The principal limitation is that adequate drug delivery depends on the patient’s ability to generate sufficient pressure drop and airflow through the device’s internal resistance to allow effective aerosolization. Patients with impaired inspiratory capacity, including some patients with severe COPD or frailty, may generate insufficient inspiratory flow for optimal drug delivery [33].

Since their introduction in the late 1960s, numerous DPI systems have been developed with substantial variation in internal design and dose-storage mechanisms. DPIs are commonly categorized into three major groups: unit-dose, multi-dose blister, and multi-dose reservoir devices [34]. Unit-dose DPIs require insertion of an individual capsule before each use, with aerosolization occurring after capsule puncture during inhalation. These devices are mechanically simple and allow visual confirmation that the capsule has been emptied, although they are less convenient due to the multiple handling steps required [35].

Multi-dose blister DPIs contain several pre-metered doses stored within individual blisters, with device actuation preparing a single dose for inhalation. These systems often incorporate dose counters and are generally more convenient than unit-dose DPIs. Multi-dose reservoir DPIs store bulk powder within an internal chamber, from which individual doses are metered during actuation. Multi-dose DPIs are compact and practical for maintenance therapy, although their performance may be affected by environmental humidity and handling conditions, depending on device design [35].

In vitro studies of contemporary DPIs have generally demonstrated acceptable dose uniformity throughout inhaler life, although the sensitivity to inspiratory flow varies substantially among devices. Comparative testing has shown that some multidose DPIs maintain relatively stable delivered doses across tested flow rates of 30–90 L/min, whereas others show greater dose reduction at lower flow rates [36].

### 6.3. Soft Mist Inhalers (SMIs)

The Respimat soft mist inhaler (SMI), currently the only widely available commercial SMI platform, is a propellant-free device that uses stored spring energy to force liquid medication through two microscopic channels, producing two opposing liquid jets that collide to generate a fine aerosol mist. This mechanism provides reproducible spray-volume delivery throughout the intended cartridge life. Studies of the reusable Respimat platform have similarly demonstrated consistent delivered-dose performance and fine-particle characteristics across repeated cartridge replacements [37]. The resulting aerosol is characterized by low plume velocity, prolonged spray duration, and a high fine particle fraction. This profile reduces oropharyngeal impaction and enhances pulmonary deposition [38].

Although the need for precise hand–breath coordination during the breath cycle is reduced compared with pMDIs, the device still requires a degree of manual dexterity during initial cartridge insertion and mechanical priming before each use.

## 7. Inhaler Selection

Although inhaler devices vary in deposition profiles and delivery mechanics—and the active pharmaceutical ingredients paired with them differ in half-life and potency—major clinical guidelines primarily organize treatment recommendations according to therapeutic drug class and combination, while inhaler device selection is considered separately.

Current guidelines for initiating inhaled therapy in obstructive lung disease emphasize treatment according to symptom burden, exacerbation risk, and disease-specific characteristics rather than a uniform sequence of escalation. For asthma, the Global Initiative for Asthma (GINA) recommends ICS-containing therapy for all adults and adolescents. In the preferred Track 1 strategy, as-needed low-dose ICS–formoterol is used in patients who do not require regular maintenance treatment, whereas maintenance ICS–formoterol combined with the same inhaler for symptom relief, termed maintenance-and-reliever therapy (MART), is used when regular controller therapy is required. Additional controller and biologic therapies may be considered for patients with uncontrolled or severe asthma [23].

For COPD, GOLD 2026 recommendations distinguish initial treatment according to the GOLD A/B/E patient groups, based on symptom burden and recent exacerbation history. Group A patients, who have a lower symptom burden and no moderate or severe exacerbations in the previous year, are generally treated with a bronchodilator, whereas LABA/LAMA combination therapy is recommended for Group B patients with greater symptom burden and for Group E patients with one or more moderate or severe exacerbations in the previous year. In Group E patients with blood eosinophil counts ≥300 cells/µL, initial LABA/LAMA/ICS triple therapy may be considered. Subsequent treatment is guided by persistent dyspnea or exacerbations, together with blood eosinophil counts when considering ICS-containing therapy [22].

The evidence base supporting drug treatment in asthma and COPD is extensive. However, few randomized trials have been designed to perform head-to-head comparisons of different inhaler device types while keeping the active drug or drug combination comparable between groups. Isolating the independent effect of the device is methodologically challenging because devices may differ concurrently in formulation, particle size distribution, and dosing. Indirect comparisons of randomized trials have shown no clinical advantage of alternative devices over standard pMDIs for ICS delivery or SABA delivery in asthma [39]. More contemporary systematic reviews similarly report no clinically meaningful differences between pMDIs and non-pMDI devices in asthma and COPD across spirometric outcomes, exacerbation rates, reliever use, quality-of-life measures, or major safety endpoints [40].

Taken together, available comparative evidence has not demonstrated consistent clinical superiority of one inhaler device class over another when comparable pharmacologic therapy is delivered appropriately. However, this population-level comparability does not imply that devices are interchangeable for every patient, as clinically important differences may emerge according to individual patient characteristics. Randomized controlled trials and treatment guidelines provide broad guidance but offer less precision for individual device selection. Achieving optimal long-term outcomes therefore requires careful evaluation of patient-specific variables that may favor one device or active drug over another.

The following sections therefore focus on identifying these patient-specific factors and clarifying how device selection can be individualized in clinical practice, with a summary of key considerations provided in Table 4.

### 7.1. Assessing Peak Inspiratory Flow Rate (PIFR) and Inspiratory Pressure

For dry powder inhalers (DPIs), adequate inspiratory effort is required to ensure proper powder deaggregation and effective lung deposition. Peak inspiratory flow rate (PIFR) has traditionally been used as a practical surrogate for this requirement, with threshold values (typically 30–60 L/min depending on device) commonly applied to assess suitability (Table 5) [41]. In comparative testing of individual DPI platforms, the proportion of patients achieving device-specific optimal PIFR varied substantially among devices, highlighting that inspiratory requirements depend on the characteristics of the specific inhaler rather than resistance classification alone [42].

However, this flow-centric paradigm does not fully reflect the underlying aerosol physics governing DPI performance. As emphasized by Clark et al., the fundamental determinant of effective powder dispersion is the energy generated across the inhaler, which is more directly related to the maximum inspiratory mouth pressure produced during inhalation [44]. The pressure a patient can generate is thus directly related to their inspiratory muscle strength. For a given inhaler, maximum flow (Q) across an inhaler relates to pressure drop (ΔP) and device resistance (R) roughly as:

$$\sqrt{\Delta P}=Q\times R$$

From this perspective, inspiratory pressure serves as the driving force for turbulent energy and particle deaggregation, whereas PIFR represents the resulting airflow generated across the device resistance. The clinical implications of suboptimal PIFR remain well recognized. Inadequate inspiratory flow has been associated with reduced bronchodilator response, impaired lung-volume improvement, and increased risk of COPD-related readmissions [41,45].

The ability to generate adequate PIFR is influenced by both patient- and disease-related factors. Lower PIFR has been associated with advancing age, female sex, shorter stature, reduced handgrip and inspiratory muscle strength, impaired lung function, hyperinflation, and greater COPD severity [46]. These associations likely reflect, at least in part, the pressure-generating capacity of the inspiratory muscles and the lung volume available for inspiration. Reduced inspiratory capacity and an increased residual volume/total lung capacity ratio, reflecting greater lung hyperinflation, have been associated with suboptimal PIFR [47]. PIFR may decline further during an acute exacerbation, potentially reflecting exacerbation-related lung hyperinflation [48]. These factors should therefore be considered when evaluating whether an individual patient can reliably generate sufficient inspiratory effort for a selected DPI.

PIFR can be measured in the ambulatory setting using handheld inspiratory flow devices such as the In-Check DIAL Inspiratory Flow Meter, which can simulate the internal resistance of different DPIs [49]. Importantly, intermediate PIFR values should be interpreted in the context of the specific device. Patients with PIFR between approximately 30 and 60 L/min should not be automatically excluded from DPI use, as lower flow rates may still generate an adequate pressure drop through higher-resistance devices. Thus, a PIFR of approximately 30 L/min may be acceptable in selected device–patient pairings. When formal PIFR measurement tools are unavailable, clinical markers such as frailty and reduced grip strength have been associated with impaired PIFR [50,51].

DPIs differ in their intrinsic airflow resistance, but resistance category alone is an incomplete guide to the inspiratory effort required for effective drug delivery. The airflow generated through a given device reflects the interaction between patient-generated inspiratory pressure and device resistance, while powder deaggregation is additionally influenced by device-specific internal geometry and turbulence generation. Consequently, PIFR should be interpreted in relation to the individual inhaler rather than extrapolated across resistance categories. Some lower-resistance devices require relatively high inspiratory flow rates, whereas higher-resistance devices may achieve their intended operating conditions at lower flow rates; therefore, resistance classification alone does not determine whether a particular DPI is suitable for an individual patient [43,44].

If inspiratory capability is insufficient for a selected DPI, alternative delivery systems should be considered. Pressurized metered-dose inhalers (pMDIs), particularly when used with a spacer or valved holding chamber, do not rely on high inspiratory flow for drug dispersion. Nebulized therapy may also provide a practical alternative in selected patients.

### 7.2. Pneumonia Risk and Inhaled Corticosteroid (ICS) Selection in COPD

Patients with COPD have a higher baseline risk of pneumonia. Exacerbations are often infection-triggered and recurrent infections are common. A large meta-analysis of clinical trial data demonstrated that ICS use significantly increased pneumonia risk in COPD (OR, 1.43; 95% CI, 1.34–1.53) [52].

However, ICS also significantly reduces the risk of acute exacerbations in appropriately selected patients. Studies of single-inhaler triple therapy demonstrate that the benefit of ICS for exacerbation prevention increases with higher blood eosinophil counts [53]. Contemporary GOLD guidelines incorporate blood eosinophil counts as a biomarker to estimate the likelihood of benefit from ICS-containing therapy. An eosinophil count ≥ 300 cells/µL may support consideration of initial triple therapy in Group E. During follow-up, patients who continue to experience exacerbations despite LABA/LAMA may benefit from escalation to triple therapy at eosinophil counts ≥ 100 cells/µL, with the expected benefit from ICS generally increasing with higher eosinophil counts [22]. Thus, the balance between exacerbation prevention and pneumonia risk depends substantially on the underlying COPD phenotype and the anticipated benefit from ICS therapy.

Patient-specific susceptibility should also be considered when assessing pneumonia risk. GOLD identifies active smoking, age ≥ 55 years, a history of prior exacerbations or prior pneumonia, body mass index (BMI) < 25 kg/m^2^, greater symptom burden, and more severe airflow obstruction as factors associated with increased pneumonia risk [22]. In addition, a large meta-analysis demonstrated a dose-dependent increase in pneumonia risk with ICS, with greater risk among patients with more severe COPD. The increased risk was observed across treatment durations (<6 months and ≥6 months), age groups, and BMI categories [52].

Pneumonia risk also appears to differ among individual ICS molecules. Large observational analyses have repeatedly found higher pneumonia risk with fluticasone than with budesonide [54]. In a large health insurance database study, fluticasone was associated with a particularly elevated and dose-related risk of pneumonia (RR, 2.01; 95% CI, 1.93–2.10), whereas the increase associated with budesonide was smaller (RR, 1.17; 95% CI, 1.09–1.26) [55]. These intraclass differences are supported by a meta-analysis of randomized controlled trials, in which the risk of serious pneumonia was significantly increased with fluticasone but not with budesonide or beclometasone [52]. Current evidence therefore primarily supports pneumonia risk as an ICS-related effect influenced by molecule, dose, and patient susceptibility, while evidence for an independent device-specific effect remains limited.

Taken together, a pragmatic strategy for ICS use in COPD is to first confirm an appropriate indication, based on exacerbation history, blood eosinophil count, and the presence of concomitant asthma features. Pneumonia risk can then be mitigated through careful patient selection, use of the lowest effective ICS dose, and consideration of molecule-specific risk, particularly in patients with multiple pneumonia risk factors. Continued ICS use should be reassessed if pneumonia occurs, with de-escalation considered when the expected benefits no longer outweigh the risks.

### 7.3. Targeting Small Airway Disease

Asthma and COPD are highly heterogeneous diseases. Although patients may share similar symptoms and airflow limitation, inflammation and obstruction are distributed unevenly throughout the bronchial tree. In a substantial subset of patients, pathology predominantly involves the small airways—the distal bronchioles with internal diameters < 2 mm [56]. Functional respiratory imaging has increasingly validated the clinical importance of this compartment, demonstrating that small airway dysfunction contributes meaningfully to symptoms, air trapping, and disease progression [57]. In the ATLANTIS study, patients with asthma underwent comprehensive assessment for small airway disease. Small airway dysfunction identified using impulse oscillometry was found to be independently associated with an increased risk of future asthma exacerbations, even among patients with otherwise well-controlled disease [58].

Conventional inhaled therapies can reach the peripheral airways, but deposition within this compartment may be proportionally lower than in the central airways. Larger aerosol particles are more susceptible to inertial impaction and sedimentation in the proximal bronchial tree, reducing the fraction available to penetrate distally. In obstructive airway disease, airway narrowing, mucus accumulation, and heterogeneous ventilation may further limit aerosol delivery to affected peripheral regions. Many conventional formulations generate aerosols predominantly within the approximately 2–5 μm range, whereas extrafine formulations are specifically engineered to generate aerosols with substantially smaller particle size [30,59,60]. As a result, these formulations may theoretically achieve greater penetration into the distal airways in patients with small airway disease.

Clinically, this approach may be most relevant in patients with suspected or documented small airway dysfunction, such as abnormal oscillometry indices, radiographic or physiological evidence of air trapping or hyperinflation, disproportionate symptoms relative to spirometry, or recurrent exacerbations despite apparently adequate therapy [61].

Trials have demonstrated the efficacy of extrafine formulations in obstructive airway disease. For example, in COPD, imaging and physiological studies have shown that switching from non-extrafine ICS/LABA therapy to extrafine triple therapy improves distal airway indices and leads to clinically meaningful improvements in symptom burden and quality-of-life scores [62]. Similar findings have been reported in asthma. Imaging-based studies have shown that treatment with extrafine beclometasone/formoterol increases small airway volume and reduces airway resistance, with structural improvements correlating with better asthma control [63].

However, these studies have important limitations. First, they were not specifically designed to enroll patients with confirmed small airway disease. In addition, relatively few trials directly compare extrafine and non-extrafine formulations while maintaining equivalent active ingredients and delivered doses. As a result, the extent to which improved outcomes are attributable specifically to particle size and its interaction with small airway dysfunction remains uncertain. Consistent with this, a large meta-analysis comparing extrafine beclometasone/formoterol with conventional ICS/LABA therapy in asthma found no significant differences in lung function, fractional exhaled nitric oxide (FeNO), or asthma control scores [64].

Overall, while extrafine formulations represent a biologically plausible strategy for targeting distal airway disease, whether specifically selecting these formulations for patients with confirmed small airway dysfunction translates into additional clinical benefit remains uncertain and deserves further study.

### 7.4. Improving Adherence with Once-Daily Dosing

Another important difference among inhaled therapies lies in dosing frequency, which is largely determined by the pharmacologic properties and duration of action of the active pharmaceutical ingredient. Some medications can be administered once daily, whereas others require twice-daily dosing to maintain adequate efficacy.

Across multiple study designs—including electronic inhaler monitoring and large administrative claims analyses—once-daily regimens have generally demonstrated higher adherence than twice-daily therapies. In electronically monitored cohorts, once-daily users showed higher median daily adherence in both asthma and COPD populations [65]. Similarly, a real-world database analysis demonstrated greater 12-month persistence, defined as continuation of treatment over time, and a lower likelihood of treatment discontinuation with once-daily regimens [66]. These findings are consistent with patient-reported preferences for simpler dosing schedules and reduced treatment burden.

Medication adherence is clinically relevant, as poorer adherence to inhaled therapy is associated with an increased risk of exacerbations in both COPD and asthma. Meta-analyses have reported approximately 40% higher odds of COPD exacerbations with poor adherence and approximately 89% higher odds of severe asthma exacerbations [67,68].

Improved adherence with once-daily therapy may therefore translate into better clinical outcomes. In an observational active-comparator study comparing two once-daily agents with a twice-daily agent, initiation of once-daily inhaled therapy was associated with a lower risk of moderate-to-severe exacerbations [69]. However, these findings should be interpreted cautiously because the once-daily and twice-daily groups received different active ingredients. The observed reduction in exacerbation risk may reflect differences in adherence associated with dosing frequency, intrinsic pharmacologic differences between the agents, device-related differences, or a combination of these factors.

### 7.5. Minimizing Multiple Inhalers and Differing Inhalation Techniques

Once-daily maintenance regimens offer a clear adherence and convenience advantage. However, most once-daily therapies are delivered via DPIs or SMIs, while short-acting rescue bronchodilators are commonly delivered by pMDI. For patients whose treatment regimen requires a separate reliever inhaler, breakthrough symptoms may therefore require use of a device with a different inhalation technique. Although rapid-acting agents may be available as a DPI in some regions, reduced peak inspiratory flow during an acute exacerbation may compromise drug delivery from some DPIs [41].

Using a pMDI and DPI properly requires substantially different techniques. This means that patients who use a non-pMDI as maintenance therapy but still require frequent rescue medication use have to master two fundamentally different inhaler techniques. Real-world studies consistently demonstrate that inhaler handling errors are highly prevalent even when using a single device type [70]. This issue has also been shown to be associated with clinically significant outcomes. In a matched cohort study comparing patients prescribed maintenance and reliever therapy using the same inhaler device type versus those prescribed different device types, patients in the similar-device cohort experienced a significantly lower rate of exacerbations (RR 0.82, 95% CI 0.80–0.84) and were less likely to require higher doses of rescue agents (OR 0.54, 95% CI 0.51–0.57) [71]. For patients who require frequent rescue medication use, simplifying inhaler technique may therefore offer meaningful advantages.

This principle aligns with both COPD and asthma guidance [22,23]. In asthma, this concept is incorporated directly into ICS–formoterol maintenance-and-reliever therapy (MART). Unlike regimens in which an ICS-based inhaler is used for maintenance with a separate short-acting β_2_-agonist (SABA) for symptom relief, MART uses the same ICS–formoterol inhaler for both scheduled treatment and symptom relief, thereby eliminating the need for a separate reliever inhaler. The rapid onset of formoterol permits its use for symptom relief, while each reliever dose also delivers additional ICS during periods of worsening asthma. This strategy offers several practical advantages by reducing reliance on SABA-only rescue therapy, using a single inhaler device for both maintenance and relief, and ensuring that increased reliever use is accompanied by additional ICS exposure.

Comparative clinical trials demonstrate that this strategy reduces exacerbation risk. In the 3335-patient COMPASS trial, budesonide/formoterol used for both maintenance and relief reduced severe exacerbation rates compared with fixed-dose salmeterol/fluticasone or fixed-dose budesonide/formoterol, each used with a separate short-acting reliever [72]. More recently, an individual-patient meta-analysis of 4863 patients with poorly controlled asthma found that switching to MART reduced the risk of severe exacerbation by approximately 29–30% compared with maintenance ICS/LABA therapy with SABA relief [73]. Thus, in appropriately selected patients with asthma, use of a single ICS–formoterol inhaler can reduce both treatment complexity and exacerbation risk.

Formoterol is also available as a maintenance LABA in COPD, and maintenance-and-reliever therapy with budesonide/formoterol has been investigated in this population. In a small randomized trial of 195 patients, budesonide/formoterol MART produced a similar rate of moderate-to-severe exacerbations compared with fixed-dose fluticasone/salmeterol plus salbutamol, while resulting in lower total daily ICS exposure [74]. However, evidence remains limited, and current GOLD recommendations support short-acting bronchodilators for acute symptom relief rather than maintenance formoterol-containing inhalers as rescue therapy. Thus, patients receiving formoterol-based maintenance therapy for COPD generally still require a separate rescue inhaler.

### 7.6. Environmental Impact of Inhaler Devices

The environmental impact of inhaler devices is primarily driven by their life-cycle greenhouse gas emissions, with the dominant contributor being propellant pMDIs. Early CFC propellants were associated with substantial environmental impact, particularly because of their ozone-depleting effects. Modern pMDIs use HFA propellants, which do not have the same ozone-depleting potential; however, HFAs remain potent greenhouse gases, and their carbon footprint generally remains higher than that of dry powder inhalers [75].

Typical estimates place the carbon footprint of a single pMDI at approximately 10–30 kg CO_2_-equivalent (CO_2_e); in comparison, DPIs generally range from 0.3–1.3 kg CO_2_e per device, while SMIs have been estimated at approximately 0.8 kg CO_2_e per device [76]. These estimates vary according to the specific device, formulation, use pattern, and life-cycle assessment methodology. From a systems perspective, inhalers contribute a notable proportion of healthcare emissions. In the United Kingdom, pMDI use alone accounts for an estimated 3.9% of the National Health Service carbon footprint [77]. Multiple studies have consistently demonstrated that switching from higher-emission inhalers, particularly pMDIs, to lower-carbon alternatives such as DPIs or SMIs can result in substantial cumulative reductions in greenhouse gas emissions [77,78,79]. Ongoing technological advances and the development of next-generation propellants are expected to reduce the greenhouse gas impact of pMDIs [80].

Although DPIs generally have a substantially lower carbon footprint than pMDIs, their environmental impact is still influenced by device manufacturing and packaging. Different DPI platforms also differ in their overall carbon footprint. Single-dose capsule-based DPIs may have relatively low carbon emissions because these devices are mechanically simpler and contain fewer plastic components. Some capsule-based systems also allow the inhaler device to be reused across multiple medication refills. Conversely, multi-dose reservoir-based and multi-dose blister DPIs require more complex internal mechanisms and greater quantities of manufacturing materials, resulting in higher carbon footprints. These devices are also typically designed around fixed-duration treatment courses and often require replacement every one to two months [76].

While transitioning to lower-carbon devices offers meaningful environmental benefits, inhaler selection should remain individualized. When clinically appropriate and when no patient-specific factors favor one device over another, preferential selection of lower-carbon devices represents a practical approach to reducing overall environmental burden.

### 7.7. Cost and Accessibility

Access and affordability are also important practical determinants of inhaler selection, since the most clinically appropriate therapy provides little benefit if it cannot be obtained or used consistently. Access to inhaled therapies varies substantially across regions and may be particularly limited in low- and middle-income countries (LMICs). Although the World Health Organization has proposed a target of at least 80% availability for essential medicines, a systematic review across 60 LMICs found that only a minority of countries achieved this threshold for short-acting bronchodilators, with even lower availability for inhaled corticosteroids (ICS) and no countries achieving this target for ICS–LABA combinations [81].

Affordability may remain a substantial barrier even when medications are available. Across LMICs, a one-month supply of short-acting β_2_-agonists has been estimated to cost approximately 1–4 days’ wages, while ICS therapy may require approximately 2–7 days’ wages, with substantially greater costs reported for some combination and long-acting inhalers [81]. Cost-related barriers are also relevant in high-income settings. In the United States, higher out-of-pocket costs for inhaled therapies have been associated with reduced medication use, cost-related non-adherence, and greater risk of exacerbations and acute healthcare utilization [82].

Cost differences between inhaler devices and formulations may also influence treatment choice. Older generic pMDI or DPI formulations are often less expensive, whereas newer single-inhaler combination therapies, particularly single-inhaler triple therapy, may have higher acquisition costs [83]. However, single-inhaler triple therapy can reduce treatment burden by simplifying the regimen and reducing device-handling complexity, which may partly offset higher medication costs through reduced healthcare utilization [84]. Accordingly, inhaler selection should consider not only pharmacologic efficacy and device characteristics but also local availability, formulary or reimbursement restrictions, and the patient’s ability to sustain treatment costs.

## 8. Conclusions

The clinical success of inhaled therapy depends on the interaction between the active pharmacological ingredient, inhaler design, and the patient’s technique and adherence. While clinical trials have demonstrated broad efficacy across platforms, this review underscores that meaningful differences remain in particle size, lung deposition, device resistance, dosing frequency, and safety profiles. Each drug–device combination carries specific advantages, limitations, and practical nuances that allow clinicians to tailor therapy to the individual patient.

Optimal inhaler selection requires matching device characteristics to patient-specific factors. These include the presence of small airway disease, peak inspiratory flow capacity, pneumonia risk when considering ICS, preference for once- versus twice-daily dosing, and the need for simple, consistent rescue strategies. Additional practical considerations such as cost, medication accessibility, and environmental impact may influence device selection in real-world practice, particularly when multiple clinically equivalent options are available.

As the therapeutic landscape continues to evolve and inhaler technology advances, the emphasis should shift from selecting “the best inhaler” to selecting the most appropriate inhaler for a given individual. This personalized approach should balance drug and formulation characteristics, aerosol performance, inspiratory capacity, coordination and inhaler technique, dosing frequency, adherence, safety, affordability, and environmental impact. Ultimately, however, the suitability of any drug–device combination should be judged by whether it achieves effective and sustainable clinical outcomes for the individual patient.

## Figures and Tables

**Table 1:** Aerosol sizes and Factors Affecting Deposition.

Particle Size Range	Deposition Mechanism	Primary Site of Deposition	Influencing Factors
>5 μm	Inertial Impaction	Oropharynx Large Airways	High flow rates Airway bifurcations
1–5 μm	Gravitational Sedimentation	Small Airways Bronchioles	Breath-holding time Particle density
<1 μm	Brownian Diffusion	Alveoli	Particle dwell time Respiratory rate

**Table 2:** Reported Lung and Extrapulmonary Deposition for Selected Inhaler Devices.

Device Category	Total Lung Deposition (%)	Extrapulmonary Deposition (%)	Ref.
Traditional pMDI	21%	55%	[16]
pMDI with valved holding chamber	52%	4%	[17]
Standard jet nebulizer	3%	33.9%	[18]
Respimat SMI	37–53%	45–56%	[16]
Aerosphere pMDI	34.5–37.7%	62–65.1%	[19]
Ellipta DPI	20.8–22.7%	Not available	[20]

Note: Values are derived from separate studies using differing assessment methods and should not be interpreted as direct head-to-head comparisons of device performance. Ellipta lung deposition was estimated using functional respiratory imaging (FRI), whereas deposition for the other devices was assessed using in vivo gamma scintigraphy. Commercial device names are provided to identify the specific inhaler platforms studied; availability and brand names may vary by country or region.

**Table 3:** Comparison of Major Inhaler Device Classes: Advantages, Limitations, and Patient Factors Influencing Selection [22,23,24,25].

Device	Advantages	Limitations	Patient Factors that Favor Use
pMDI	Widely available Low dependence on inspiratory flow Spacer can reduce coordination requirements	Requires slow inhalation and actuation–inhalation coordination when used without spacer Technique errors common Environmental impact associated with propellant use Dose uniformity of suspension pMDIs depends on adequate shaking before each actuation	Limited inspiratory flow Able to coordinate actuation and inhalation or use a spacer Frequent pMDI rescue use when consistency of inhaler technique is preferred
DPI	Breath-actuated Avoids actuation–inhalation coordination Propellant-free	Requires adequate inspiratory effort Inspiratory requirements vary with device resistance and design Dose uniformity of DPIs may depend on adequate inspiratory effort and proper device handling and may be affected by humidity.	Adequate inspiratory ability Difficulty coordinating pMDI actuation with inhalation
SMI	Slow-moving prolonged aerosol Low dependence on inspiratory flow Lower hand-breath coordination demands than pMDI	Requires device preparation and some coordination Fewer available drug/device combinations	Limited inspiratory flow Difficulty generating forceful DPI inhalation Difficulty with hand-breath coordination

Note: Characteristics are generalized across inhaler classes; substantial differences exist among individual devices and formulations. Device selection should therefore be individualized according to medication availability, inspiratory ability, coordination, dexterity, cognition, patient preference, and correct device use.

**Table 4:** Summary of Factors Relevant to Individualized Inhaler Selection.

Patient-Specific Factor	Clinical Implication	Applicability	Suggested Selection Strategy
Inspiratory Flow/ Pressure Capability	Suboptimal inspiratory pressures and PIFR limit DPI performance	Both	Low PIFR: prefer pMDI (with spacer) or SMI; Adequate PIFR: DPI acceptable
High pneumonia risk	Increased risk of ICS-associated pneumonia	COPD	When ICS is indicated, consider pneumonia risk, ICS dose, and potential differences among ICS molecules
Small airway disease	Potential benefit from enhanced peripheral deposition	Both	Consider extrafine particle formulations in selected patients
Adherence (dosing frequency)	Complex regimens reduce adherence	Both	Prefer once-daily agents when possible
Frequent reliever use	May indicate inadequate disease control Risk of increased complexity of multidrug/device regimens	COPD	Minimize the number of inhalers and differing device types when feasible
		Asthma	Consider ICS–formoterol-based reliever strategies when appropriate
Hand-Breath coordination	Poor coordination reduces pMDI effectiveness	Both	Prefer DPI or SMI Use spacer if pMDI required
Cost/access constraints	Limits real-world implementation	Both	Align device choice with available and affordable options
Environmental considerations	Propellant-related carbon footprint	Both	Consider DPI or SMI over pMDI when clinically appropriate

**Table 5:** Common DPI Platforms, Inspiratory Resistance, and Recommended PIFR [41,43].

Device	Inspiratory Resistance (kPa^0.5^ (L min^−1^)^−1^)	Minimal PIFR (L/min)	Optimal PIFR (L/min)
Breezhaler	0.017	50	50
Diskus/Accuhaler	0.027	30	60
Ellipta	0.027	30	60
NEXThaler	0.036	35	35
Turbuhaler	0.039	30	60

## Data Availability

No new data were created or analyzed in this study. Data sharing is not applicable to this article.

## Abbreviations

The following abbreviations are used in this manuscript:

BMI	Body Mass Index
CFC	Chlorofluorocarbon
CO_2_e	Carbon dioxide equivalent
COPD	Chronic obstructive pulmonary disease
DPI	Dry powder inhaler
FeNO	Fractional exhaled nitric oxide
FPF	Fine particle fraction
FRI	Functional respiratory imaging
GINA	Global Initiative for Asthma
GOLD	Global Initiative for Chronic Obstructive Lung Disease
HFA	Hydrofluoroalkane
ICS	Inhaled corticosteroid
LABA	Long-acting β_2_-agonist
LAMA	Long-acting muscarinic antagonist
LMIC	Low- and middle-income country
MART	Maintenance-and-reliever therapy
MMAD	Mass median aerodynamic diameter
PIFR	Peak inspiratory flow rate
pMDI	Pressurized metered-dose inhaler
SABA	Short-acting β_2_-agonist
SAD	Small airway disease
SMI	Soft mist inhaler
VHC	Valved holding chamber

## Appendix A

**Table A1:** Representative Commercially Available Inhaler Devices and Formulation Characteristics.

Device Class	Device Platform-Representative Product	Active Ingredient(s)	Delivery System	Reported MMAD (µm)	Reference
pMDI	Modulite—Foster/Fostair	Beclometasone dipropionate Formoterol	Solution pMDI	1.4	[85]
pMDI	Modulite—Trimbow	Beclometasone dipropionate Formoterol Glycopyrronium	Solution pMDI	1.1	[86]
pMDI	Alvesco	Ciclesonide	Solution pMDI	1	[87]
pMDI	Aerosphere—Bevespi	Glycopyrronium Formoterol	Co-suspension pMDI	Glycopyrronium 3.0 Formoterol 3.1	[88]
pMDI	Aerosphere —Breztri/Trixeo	Glycopyrronium Formoterol Budesonide	Co-suspension pMDI	2.51–2.53	[89]
pMDI	Evohaler—Ventolin	Salbutamol	Suspension pMDI	2.8	[85]
pMDI	Evohaler—Seretide	Fluticasone propionate Salmeterol	Suspension pMDI	2.7	[90]
pMDI	Rapihaler—Symbicort	Budesonide Formoterol	Suspension pMDI	Budesonide 3.6 Formoterol 3.3	[91]
pMDI	Autohaler—QVAR	Beclometasone dipropionate	Breath-actuated ^1^ Solution pMDI	1.26	[92]
DPI	Diskus/Accuhaler—Seretide	Fluticasone propionate Salmeterol	Pre-metered multidose blister DPI	3.3–3.5	[93]
DPI	Turbuhaler—Symbicort	Budesonide Formoterol	Multidose reservoir DPI	Budesonide 2.0–2.3 Formoterol 2.5–2.7	[94]
DPI	Easyhaler—Bufomix	Budesonide Formoterol	Multidose reservoir DPI	Budesonide 2.2–2.5 Formoterol 2.7–3.0	[94]
DPI	NEXThaler—Fostair	Beclometasone dipropionate Formoterol	Breath-actuated ^1^ multidose reservoir DPI	Beclometasone 1.5 Formoterol 1.4	[95]
DPI	Spiromax—DuoResp	Budesonide Formoterol	Multidose reservoir DPI	Budesonide 2.1–2.4 Formoterol 2.1–2.4	[95,96]
DPI	Genuair/Pressair—Eklira/Tudorza	Aclidinium	Multidose reservoir DPI	2.4	[97]
DPI	Breezhaler—Onbrez/Arcapta	Indacaterol	Single-dose capsule DPI	2.6–3.5	[98]
DPI	Breezhaler—Seebri	Glycopyrronium	Single-dose capsule DPI	2.8	
DPI	Breezhaler—Ultibro	Indacaterol Glycopyrronium	Single-dose capsule DPI	Formulation-specific MMAD not reported ^2^	[99]
DPI	Breezhaler—Enerzair	Mometasone Indacaterol Glycopyrronium	Single-dose capsule DPI	Formulation-specific MMAD not reported ^2^	[99]
DPI	HandiHaler —Spiriva	Tiotropium	Single-dose capsule DPI	3.8–5.8	[100]
DPI	Ellipta—Relvar/Breo	Fluticasone furoate Vilanterol	Pre-metered multidose blister DPI	Fluticasone 3.2 Vilanterol 1.8	[97]
DPI	Ellipta—Anoro	Umeclidinium Vilanterol	Pre-metered multidose blister DPI	Formulation-specific MMAD not reported ^2^	
DPI	Ellipta—Trelegy	Fluticasone furoate Umeclidinium Vilanterol	Pre-metered multidose blister DPI	3.76–3.87	[89]
SMI	Respimat—Spiriva	Tiotropium	Mechanically generated aerosol	3.3	[101]
SMI	Respimat—Striverdi	Olodaterol	Mechanically generated aerosol	3.7	[101]
SMI	Respimat—Spiolto/Stiolto	Tiotropium Olodaterol	Mechanically generated aerosol	Combination 3.4	[101]

MMAD values were obtained from separate published sources and should not be interpreted as direct head-to-head comparisons. Reported values may vary according to formulation, measurement methodology, device operating conditions, and inspiratory flow rate. ^1^ Actuation is triggered automatically by inhalation, eliminating the need for manual actuation. ^2^ MMAD data are available only for the individual components studied separately.
